# The Diagnosis and Treatment of Chronic Bright’s Disease

**Published:** 1911-07

**Authors:** Lewis M. Gaines

**Affiliations:** Atlanta, Ga.; Professor of Physiology, Atlanta School of Medicine


### THE DIAGNOSIS AND TREATMENT OF CHRONIC BRIGHT'S DISEASE.

By Lewis M. Gaines, B.S., M.D., Atlanta, Ga.

*Professor of Physiology, Atlanta School of Medicine.*

Bright's Disease is on the increase in America. There are more cases of this dread malady in our midst than ever before. Often exceedingly insidious in its onset, before the luckless victim is aware its destructive processes have made such progress that little can be accomplished by the physician. If the disease can be recognized in its incipiency, however, much may be done to prolong life, and that in comparative comfort and activity.

It is true that chronic Bright's Disease is incurable from the very nature of the anatomical lesions which are present. The disease is simply the manifestation of a destructive process in the kidney. But, while the destruction cannot be repaired and new, healthy tissue induced to grow, the process may often be arrested more or less completely, and a very great deal done to relieve the overtaxed organism.

### Types of the Disease.

Two main types of chronic Bright's Disease occur. These types are each characterized by certain striking differences in the changes which occur in the kidney itself, in the pathological variations of the urine, and in the symptoms and physical signs. Not infrequently these types approach each other closely, but the division is necessary and helpful.

### Type I.

In the Parenchymatous type of the disease, the kidney is large, and white in color. The urine is more or less diminished in quantity, of normal or high specific gravity, often of a dirty yellowish color, and turbid. Upon examination, a large amount of albumin is found, and a diminished urea excretion. Microscopically, hyaline and granular casts in abundance are seen. Sometimes fatty or waxy casts occur, and rarely epithelial or blood casts. Red blood corpuscles and leucocytes are frequent. There is often considerable granular detritus, representing degenerated epithelial cells from the kidney.

The symptoms of this type of the disease are striking, when the disease is fully developed, but the onset is frequently insidious. It may folow any of the acute infections, especially scarlet fever, diphtheria, and malarial fever. Often, after a period of failing health, characterized by loss of strength or digestive disturbances, slight puffiness of the face, particularly under the eyelids, and a peculiar pallor are noticed, shortness of breath and dropsy in the extremities appear, and the diagnosis is at once suggested.

The important thing is to examine the urine as a routine procedure in all suspicious cases, as characteristic changes are often present in it before the marked symptoms described above appear.

It is well to remember that this type is far more frequent before middle life. Not a few cases occur in children, following the diseases of childhood. Many cases occur between the ages of thirty and forty, apearing very insidiously.

TYPE II.

In the interstitial form of the disease the kidney is shrunken in size and often granular in appearance. The urine is frequently increased in amount. Often it is normal in quantity. The figures given in many text-books for the normal 24-hour amount of urine are too high. From 30 to 40 ounces (900 to 1,300 c. c.) may be considered in America an average amount of excretion for twenty-four hours. In many cases of interstitial nephritis the quantity varies from 2,000 to 3,000 c. c. In many other cases there is no such increase. More constant is the change in specific gravity, which is persistently low in practically every case of this type, and constitutes a very important diagnostic sign.

Albumin in the early stages is present only in traces, and often is absent altogether for varying periods. It is absent especially in the early morning urine. Later in the disease the albumin may become very abundant, the quantity of urine diminish, and the urine assume many of the characteristics of type I. I wish to emphasize the care which must be used in detecting slight traces of albumin. The test must be delicately performed. A four-inch test tube, scrupulously clean, and clear, filtered, slightly acid urine are essential. To the test-tube, three-fourths filled with the urine, one-sixth its volume of a saturated solution of sodium chloride should be added to rule out the precipitation of nucleo-albumin, which is without pathological significance. Then, after heating the upper part of the tube to boiling, add, drop by drop, three drops of 10% nitric acid, heat again, and add three more drops. Now hold the tube in front of the window, but against a black background. If albumin be present it will appear as a more or less faint cloud in the heated portion of the urine. The faintest possible trace often appears as a blue haze.

The microscopical examination of the urine is of great importance. The sediment is usually very scanty, but contains an occasional hyaline or finely granulated cast, a few leucocytes, or a moderate number of red blood corpuscles. Several slides may have to be carefully searched on a number of different occasions before these pathological constituents are disclosed. Sometimes the most prolonged search is unrewarded.

The symptoms of Chronic Interstitial Bright's Disease are extremely varied, when present, and all too frequently are conspicuous by their absence until the supervention of one of the serious or fatal complications. To exhaust the symptomatology would consume far more space than is allotted to this paper. I desire simply to enumerate the most frequent symptoms which have occurred in my own experience.

1. *Circulatory Disturbances,* the result of persistently high blood pressure, which is later accompanied by hypertrophy and dilatation of the heart. In this type of Bright's Disease the intelligent examination and treatment of the heart is of supreme importance. Indeed, the heart bears so close a relation to the kidney that this type may well be called *cardio-renal disease.*

These circulatory disturbances are often not noticed by the patient, but they may cause headaches, vertigo, palpitation, and digestive disturbances, for which symptoms the physician is consulted, and unless the medical man is wary, he may fail to realize the gravity of the case.

2. *Respiratory Disturbances,* most frequently manifested by attacks of shortness of breath. These attacks may come on following exertion, gradually growing more and more marked and accompanied by increasing weakness. Or, the attacks may be independent of exertion, suddenly coming on at night, waking the patient out of a sound sleep. Such an attack may be the very first indication to a vigorous man that he is not in perfect health. These attacks are often very distressing and may exactly resemble attacks of ordinary bronchial asthma. The patient sits up in bed, gasping for breath until relief comes.

3. *Disturbances of Digestion* are very common, sometimes alone, often in combination with other symptoms. These disturbances manifest themselves by simple dyspepsia and loss of appetite, or by more violent disturbances, as intense nausea and vomiting or profuse diarrhoea. The vomiting and diarrhoea are frequently compensatory phenomena, and represent Nature's effort to eliminate toxic substances. Sudden checking of either process may result disastrously. The condition calls for very careful treatment. One of my patients has an attack of intense vomiting periodically, and is much better after such an elimination.

4. *Disturbances of the Nervous System*, especially headaches and neuralgia in various peripheral nerves. Associated with these neuralgic symptoms are frequently cramps, apparently muscular. The first symptom which one of my patient had was wry neck. Prior to the occurrence of this condition she considered herself in excellent health. Tremor in another case was an early symptom.

5. *The Special Senses* are often early affected. Disturbances of vision are very common and important warnings. A wide awake oculist will first detect many early cases. A lady who recently came under my care, had, as a very early symptom, double vision, later, dimness of vision, and greatly to her discomfort most annoying disturbances of smell. The most tempting and delicious viands gave forth to her the vilest odors, so that the taking of food became an ordeal. Vertigo is by no means rare as an early symptom, and may continue for years.

6. *Skin Affections* are by no means infrequent as early or percursory symptoms, particularly eczema and dryness of the skin.

Oedema is, in this type of the disease, not a prominent feature. Very slight swelling of the ankles may be demonstrated at times, especially when the patient has been on his feet several hours. Late in the disease, oedema may become marked.

Hemorrhages are by no means infrequent. These hemorrhages may take place in various localities, and lead to erroneous diagnoses. Thus, one of my recent cases had persistent hemorrhage from the uterus. Her physician advised operation, and she went to the hospital, and was prepared. Fortunately, the surgeon recognized the condition, and she fell into my hands for medical treatment. She later had extensive bleeding from the posterior nares.

It may be seen from the foregoing that in chronic Bright's Disease we have to deal with a malady which presents itself in ever varying guise, and ever changing form. In order to correctly diagnose the condition, one should keep in mind the following

### Diagnostic Summary.

1. In every case presenting suspicious symptoms, obtain and examine at frequent intervals, a 24-hour specimen of urine.

2. A careful urinalysis, and an accurate observance of symptoms and signs will clear up the diagnosis in a large percentage of cases.

3. In the urine the findings are very different, depending upon the type of the disease. A large amount of albumin in urine of normal specific gravity is characteristic of the parenchymatous type. A trace of albumin, or even absence of albumin, together with persistent low specific gravity is characteristic of the interstitial type. The two types may, however, approach each other, especially late in the disease.

4. Examination of the heart and arteries, and the taking of the blood pressure are essential in making an accurate diagnosis of interstitial nephritis. Persistent low specific gravity of urine, with or without the presence of albumin, or an occasional cast or blood corpuscle, together with persistent high blood pressure means that interstitial changes have occurred in the kidney.

5. Interstitial nephritis of extreme grade is consistent with total absence of subjective symptoms on the part of the patient. Many cases are brought to light in the course of an examination for life insurance. If symptoms do occur, they may be very slight. Hence, especially in a patient over forty, this condition should constantly be kept in mind.

### Functional Test.

For many years there has been a constant effort to devise a reliable functional test of the kidney. Supposing the diagnosis of nephritis has been successfully made, the next question that suggests itself is concerning the amount of healthy kidney tissue left, and hence what the outlook for the future is, and what benefits may be expected from treatment.

Within the past few years a reliable functionad test has been devised by two brilliant American investigators, Rowntree and Geraghty. Six grams of *phenolsulphonephthalein* are injected into the heavy muscles of the back. In a normal individual, five to ten minutes after administration, the substance appears in the urine, and forty to sixty per cent. of the amount given is recovered in the first hour and fifteen to twenty per cent. the second hour, making in all sixty to eighty-five per cent. recovered altogether. In cases where kidney function is impaired, the drug appears only after a long interval, and in very small amount, or in other words, the diseased kidney is incapable of excreting the drug in proportion as it is diseased. Thus in two cases of chronic interstitial nephritis, less than one per cent. of the drug was recovered in the course of an hour. Both patients died of uraemia within two months. Where low grades of functional activity are demonstrated, much can often be done to stimulate activity and prolong life. It is of value to make the test from time to time, in order to be sure the kidney is kept in good condition. The drug is, of course, perfectly harmless, and its injection occasions no unpleasant symptoms. I consider this test one of the greatest contributions made in recent years to our methods of diagnosis. Dr. Geraghty has himself shown me this work in his laboratory, and I am convinced of its very great value.

### Treatment.

If good results are to be obtained in treating Bright's Disease, each patient must form a subject of special study. No hard and fast regulations can be laid down. However, there are certain general principles which constitute an invaluable guide in deciding the best course to pursue in all cases.

PRINCIPLES OF TREATMENT.

1. Arrest, or retard the degenerative process in the kidney, and maintain renal compensation, avoiding the use of all kidney irritants whether in food or drugs.

2. Diminish nitrogenous waste to such an extent that it can be dealt with by the kidney tissue which remains.

3. Counteract the baneful influence of toxic products, the result of faulty metabolism.

4. Regulate the blood pressure, and watch very closely the heart and circulation.

5. Improve the general nutrition, and do not fail to remember the immense value of adroit psychic suggestion, as this is often of the greatest importance.

METHODS OF TREATMENT.

I venture the assertion that no two patients with chronic nephritis, especially the interstitial type, can be treated exactly alike, and the best possible results obtained. Non-realization of this fact accounts for frequent failures to relieve nephritics, and the precipitation of grave or fatal complications. The first thing, therefore, to be done, is to spend a sufficient time in studying the patient, and estimating the weakness which his kidney disability has brought about.

DIET.

The majority of persons consume a large excess of nitrogenous food. The result is an excessive nitrogenous waste which must be excreted through the kidneys. The retardation of the degenerative process is best begun by giving the kidneys a rest from such overwork. It is my plan to eliminate all meat and eggs from the diet for a varying period, watching carefully the effect, particularly upon the urine, the weight and strength, and the blood pressure. During this period of restriction, vegetables, fruits and fruit juices, with cereals and stale bread are given. All indigestible articles and all fried foods are prohibited. Sweet milk, and preferably buttermilk are allowed, but in moderation. Occasionally, a strict milk diet for a limited time is advantageous. Abundance of butter and a moderate amount of digestible sweets are permitted, provided the digestion is good. Particularly in warm weather is this diet adhered to. If, however, the patient loses weight and strength, meat in moderation is allowed, especally fish, crisp breakfast bacon, fowl, mutton and beef in small amounts. It is of extreme importance to maintain a good nutrition, and a patient can be greatly injured by too zealous a regulation of his diet. When the patient can be under close supervision, it is well to determine the daily caloric value of his food, and watch the effect of his food by the functional test at intervals.

In all cases where oedema is a feature, and frequently in other conditions, no single measure is more important than the reduction of salt in the food. As a general rule, nephritic patients eliminate only about one-half of the amount of sodium chloride normally contained in the urine. This retention is a factor in the production of oedema, and probably certain other unfavorable symptoms. Hence not over 120 grams a day should be allowed. To do this food should be cooked without salt, and none used at table for seasoning. The salt naturally contained in the food taken will supply the necessities of the body, certainly for considerable periods. This method is rather hard on patients at first, as they are frequently heavy salt eaters, but they soon learn to forego it, and are glad to co-operate when they see the good results of abstinence.

Alcoholic drinks of all kinds should be absolutely prohibited. In persons who have been accustomed to consume a considerable amount of alcohol regularly it may be dangerous to suddenly cut off the alcohol, but it should be reduced as rapidly as possible.

Tea and coffee are allowable in moderation.

### WATER.

From time immemorial water has been given in various ways in the treatment of chronic Bright's Disease, and the particular virtues of many particular waters exploited. Such exploitation is done more to increase the sale of the water than to benefit the patients.

It is my belief that any pure water, preferably spring water, best serves the purpose. Owing largely to an aggressive advertising campaign, there is a very general impression that lithia water is almost a specific for the various ills to which the kidney is heir. We are told that not only will it relieve, but frequently that it will prevent nephritis. I believe a certain amount of damage is done by the abnormal stimulation the kidney tissue receives from the long continued, unbroken use of lithia water. Furthermore, several very popular brands of lithia water contain large amounts of sodium chloride, which is distinctly contraindicated in all forms of nephritis. Many patients believe the salty taste of these waters is due to the large amount of lithia contained therein. The truth is the salty taste is due to nothing else than common table salt, which in this excess is positively injurious.

Not only are these salty and highly stimulating waters injurious, but distilled water, which is frequently offered for sale in cities, is also harmful. Tyrode and others have shown that distilled water is an irritant and capable of causing marked degrees of inflammation under certain conditions. Likewise, city water which has been subjected to chemical purifying processes, may prove irritating.

I advise pure spring water, either with no mineral constituent, or with only a lithia constituent.

The amount of water a patient should take is a matter of importance. In oedema the amount should be limited as much as possible until the swelling subsides. In the polyuria of interstitial nephritis the patient demands an excess. From three to four pints daily is a good average, and forced drenching of the system with water should not be practiced.

The external application of water is of great value. In nephritis, the skin assumes added importance as a channel of excretion, and hence should be kept in the best possible condition. To this end, appropriate hydrotherapy is very necessary. A great deal can be done at home, but the best results are obtained in an institution, where baths can be scientifically administered. Under certain conditions, baths given after the Nauheim method are of great value. This method should not be tried if there is a large amount of albumin or blood in the urine, or if there is marked arterio-sclerosis.

### Rest and Exercise.

No rigid rules can be laid down concerning rest and exercise. Each patient, within certain limits, is a law to himself. Too much of either is a detriment. In advanced cases, especially with oedema and dyspnoea, prolonged rest is a necessity. In the interstitial form, particularly when compensation is good, proper exercise is invaluable.

Patients should be warned against over-exertion, particularly sudden over-exertion. A nephritic should never run to catch a street car, which is the most common form of over-exertion in cities.

When exercise can be combined with recreation, its value is enhanced. For this reason, golf in moderation is valuable. Other similarly mild recreative exercises, suited to the patient's tastes readily suggest themselves.

In this connection, it is perhaps appropriate to refer to certain phases of the psychic treatment of nephritis. Most patients with chronic diseases are singularly amenable to suggestion. The skillful physician will make use of this fact in ways too numerous to mention.

Introspection must be strongly discouraged. Examination of the patient's urine by himself must never be tolerated. I have known of several cases where intelligent patients who acquired the technic of urine examinations, were constantly testing their urine, and were constantly miserable. When they ceased the practice, improvement was prompt.

The mind must be occupied by pleasant diversions, whether by occupation or recreation, or change of scene. Travel is frequently of great value. One of my patients who had periodic attacks of severe nausea and vomiting, and was losing ground, went abroad upon my advice. A stay of several months in Italy worked wonders, the attacks ceasing entirely. Often a stay in a sanatorium is productive of immediate and prolonged improvement, partly due to the psychic factor. Frequently, the home physician is treating a case in the most approved manner without avail. Almost immediately upon change of scene, the same methods will accomplish striking results.

### Drug Treatment.

It is to be remembered in dealing with Bright's Disease that in early cases little or no medicine is required, because compensation is good, and advice is to be more especially directed along the line of diet, water drinking, what to abstain from, rest and exercise, and the re-adjustment of the daily mode of life to a basis consistent with the disability. As symptoms arise, however, the aid of drugs must be invoked.

Anaemia is frequently one of the early symptoms. For this condition one of the forms of iron is most productive of benefit. I have used for this purpose Basham's Mixture in doses of two teaspoonfuls to two tablespoonfuls after meal. The following mixture has also proved useful:

Rx. Ferri sulph. ---------------------- gr. iss
Magnesii. sulph. -------------------- ʒ i
Ac. sulph. dil. ---------------------- m x
Aq. menth pip. q. s. ------------------ ℥ i
M. Ft. Sol.
Sig. This draught in wineglass of water after meals.

In cases where hypertension and arterio-sclerosis is a feature, the iodides often prove wonderfully beneficial, if carefully given. My plan is to order one ounce of a saturated solution of potassium iodide, and a three ounce bottle of essence of pepsin. I have the patient begin with five drops of the iodide solution in a wineglass of water to which is added a teaspoonful of the essence of pepsin. This is taken after meals, and the dose of iodide gradually raised or lowered according to its action. When restlessness, insomnia, or other nervous symptoms are persistent, I order a solution of sodium bromide, 15 grains to the dram, and direct that a teaspoonful of this be added to the potassium iodide draught. This plan has proved very satisfactory.

If the appetite flags, any of the bitter tonics may be tried. I have found the following combination successful:

Rx. Tr. nucis vom. ........................ ʒ ii

Ac. hydrochlor. dil. .................... ʒ iv

Tr. Calumbae q. s. ...................... ℥ iii

M. ft. sol.

Sig. Teaspoonful in wineglass of water after meals.

It is always important to remember what drugs to avoid as well as what to use. Atropine should be used sparingly. Potassium chlorate should not be used at all. Neither such kidney irritants as cantharides, copaiba, carbolic acid, or the coal tar derivatives. Drugs which have a cumulative action, as digitalis must be used with caution.

The continued use of the diuretics is by no means advisable. The flow of urine and excretion of solids should be carefully watched, and if diminished, dieuretics should be given only until the normal flow is secured. Or, if there is increasing oedema, we should attempt its removal in part by the use of diuretics. A number of diuretics are available. Provided there is sufficient kidney tissue remaining to be acted upon, caffeine, diuretin and acet-theocin-sodium are all useful, given with sufficient water. I use a pure lithia water in abundance under these conditions, and always there should be kept a careful record of the amount of water given and the amount of urine voided in each 24 hours.

If, as is so often the case, the diminshed flow of urine is due to a weakening heart, some form of digitalis is almost indispensable. I greatly prefer for this purpose the infusion, freshly made from reliable digitalis leaves. An honest druggist is a necessity at this juncture. It is important to remember that many speciments of leaves are almost if not entirely inactive. Attention to this point may mean saving a patient's life. The results obtained in a favorable case are often almost miraculous. I order a half ounce well diluted every four hours till the flow of urine is resumed. During the administration of the digitalis the patient should be at perfect rest. If a good preparation fails to produce results in 48 hours, the prognosis is distinctly unfavorable.

The infusion accomplishes the double purpose of stimulating the heart and kidney. In some cases the patient is unable to retain the digitalis. In such cases digalen may be tried hypodermically. Digipuratum is sometimes advantageous. As a last resort for a failing heart strophanthin intravenously has accomplished most striking results.

In cases where dropsy orascites is a feature, digitalis is often administered with telling effect in the well-known Niemeyer pill.

Rx. Hydrarg. chlor. mit.
Pulv. digitalis
Pulv. scillae, a. a., gr. I
Sig. One pill every four to six hours.

Under the influence of this pill a remarkable disappearance of the fluid often takes place, and the heart regains its tonicity.

The regulation of the bowels is a very essential matter in the treatment of nephritis. In many cases there is a tendency to constipation. A saline cathartic in a large number of cases is the best remedy to use in nephritis. The following has proved a valuable combination:

Rx. Mag. sulph.
Sodii. phos. a. a. ℥ss.
Pulv. efferves. q. s.
Sig. Take in a glass of very hot water, one-half to one hour before breakfast.

The above draught taken several times a week will often relieve many of the symptoms due to retained toxines, such as nausea, loss of appetite, foul taste, and headache. Furthermore, it diminishes the tendency to oedema, and lowers the blood pressure when excessive.

If the salines become too depleting, a good preparation of cascara is useful. From a half to one teaspoonful of the aromatic fluid extract at bedtime, or half that amount after each meal will often bring relief.

For obstinate cases of constipation I have used the following pill with considerable success:

Rx. Eserine sulph. ---------------------- gr ¼
Strych. sulph. ---------------------- gr. ¼
Resina. podophyl ---------------------- gr. iiss
Ext. cascara sagrada ---------------------- gr. i
M. ft. pil No. x
Sig. One to two pills at bedtime.

In some of these obstinate cases other measures must be employed, such as abdominal massage, and special exercises. I have had success with the use of the high frequency electric current, when many other measures had failed.

The headaches so frequent in Bright's Disease are most frequently due either to retained toxines or to high blood pressure. Toxic headaches are best relieved by free elimination in the manner already indicated. If the relief is not prompt, for the pain, I prefer codeine in quarter grain doses, repeated if necessary every half hour until one or even two grains have been taken. When the headaches are due to high blood pressure, depletion with salines is usually advisable, combined with the use of nitroglycerine grain on one-hundredths every two hours. The effect of such medication should be closely watched by means of the sphygmo-manometer. I am now using the Faught apparatus with great satisfaction.

Vomiting and diarrhoea or most frequently compensatory phenomena, and must be treated with caution. Vomiting is best treated by gastric lavage. Morphine must be used with great circumspection. Diarrhoea, representing Nature's attempt to eliminate, should constitute a strong hint to further this process. Hence salines should be administered and frequent colonic irrigations with hot normal salt solution given. Often small broken doses of calomel are helpful.

The attacks of renal asthma call for immediate relief, and I have long since given up all remedies except morphine, which should be given in full doses hypodermically, and repeated as often as necessary. Much has been said concerning the harmful effects of morphine in nephritis. Provided elimination is properly kept up, I believe no harmful effects will follow the use of opium or its derivatives. In 1910 a gentleman seventy-five years of age came under my care, suffering with nightly attacks of the most urgent dyspnoea. His struggles to breathe were most distressing to witness. Morphine always afforded relief, though the attacks recurred with great frequency. He had other serious symptoms, such as insomnia, weakness and emaciation, and later an active delirium. During the course of the months he was under my care he consumed a considerable amount of morphine, yet he made a most remarkable recovery as far as symptoms were concerned, and at present has regained his weight and strength, and is leading an active life for a man of his years. Morphine apparently had no deterring effect in the effort to establish and maintain renal compensation, which at the present time is seemingly perfect in his case.

The occurrence of uraemic symptoms is of ominous signifigance, and demands prompt and energetic action. It represents the effects of retained toxines, and the most important thing in the treatment is to obtain copious bowel movements, free sweating and a copious flow of urine. In proportion as the physician is successful in these directions in just that proportion the patient improves. It may be necessary to administer the most powerful remedies for this purpose. Calomel in five grain broken doses followed by salines should be tried in the milder cases. In the more severe cases hydrogogue cathartics, such as elaterium and croton oil must be used. High colonic irrigations, and lavage of the blood after Sahli's method will sometimes tide the patient over an attack. Morphine must be used to control convulsions and delirium. For the persistent insomnia and peculiar restlessness, bromides and trional together are sometimes effective. Usually the physician must finally resort to morphine.

### Recapitulation.

1. While no hard and fast rules for treatment can be laid down, there are certain principles, which it is always helpful to keep in mind.

2. While the disease is essentially incurable, careful supervision and treatment will produce most excellent results, and often prolong life in comfort and activity for many years.

3. The most essential points in the treatment are (a) retardation of degenerative processes in the kidney, (b) maintenance of renal compensation, (c) maintenance of cardiac compensation, and a proper blood pressure level, (d) constant and free elimination through the excretory channels.

823 Candler Building.

